# Hsp90 Inhibition Decreases Mitochondrial Protein Turnover

**DOI:** 10.1371/journal.pone.0001066

**Published:** 2007-10-24

**Authors:** Daciana H. Margineantu, Christine B. Emerson, Dolores Diaz, David M. Hockenbery

**Affiliations:** 1 Clinical Research Division, Fred Hutchinson Cancer Research Center, Seattle, Washington, United States of America; 2 Human Biology Division, Fred Hutchinson Cancer Research Center, Seattle, Washington, United States of America; The Research Institute for Children, United States of America

## Abstract

**Background:**

Cells treated with hsp90 inhibitors exhibit pleiotropic changes, including an expansion of the mitochondrial compartment, accompanied by mitochondrial fragmentation and condensed mitochondrial morphology, with ultimate compromise of mitochondrial integrity and apoptosis.

**Findings:**

We identified several mitochondrial oxidative phosphorylation complex subunits, including several encoded by mtDNA, that are upregulated by hsp90 inhibitors, without corresponding changes in mRNA abundance. Post-transcriptional accumulation of mitochondrial proteins observed with hsp90 inhibitors is also seen in cells treated with proteasome inhibitors. Detailed studies of the OSCP subunit of mitochondrial F1F0-ATPase revealed the presence of mono- and polyubiquitinated OSCP in mitochondrial fractions. We demonstrate that processed OSCP undergoes retrotranslocation to a trypsin-sensitive form associated with the outer mitochondrial membrane. Inhibition of proteasome or hsp90 function results in accumulation of both correctly targeted and retrotranslocated mitochondrial OSCP.

**Conclusions:**

Cytosolic turnover of mitochondrial proteins demonstrates a novel connection between mitochondrial and cytosolic compartments through the ubiquitin-proteasome system. Analogous to defective protein folding in the endoplasmic reticulum, a mitochondrial unfolded protein response may play a role in the apoptotic effects of hsp90 and proteasome inhibitors.

## Introduction

Hsp90 is an abundant cytosolic chaperone (1–2% of cytosolic protein) involved in the turnover, trafficking and activity of a large number and variety of client proteins. These include membrane-associated and soluble protein kinases (many recognized as oncogenes) and transcription factors [1], [2]. Small molecule hsp90 inhibitors that bind to the N-terminal ATP binding pocket inhibit chaperone function. Consequently, many client proteins are targeted for degradation via the ubiquitin-proteasome pathway. In response to hsp90 inhibition, cancer cell lines exhibit several types of response, including reversal of transformation, differentiation and apoptosis [3]–[5]. Selective cytotoxicity for cancer cells is associated with expression of hsp90 client proteins, such as Bcr-Abl, FLT3 bearing an internal tandem duplication, and ErbB2, and has been suggested to reflect a survival requirement for multiple signaling pathways that depend on hsp90 chaperone function [6]–[9]. Apoptotic cell death in hsp90-inhibited cells involves mitochondrial pathways with cytosolic accumulation of cytochrome c and SMAC/Diablo [10].

As an early event in cells destined for apoptosis, hsp90 inhibitors induce mitochondrial proliferation, resulting in the accumulation of a fragmented network of mitochondria filled with homogeneous, electron-dense material obscuring the internal cristae [11], [12]. Expansion of the mitochondrial compartment is associated with reduced mitochondrial membrane potential (ΔΨ_M_), pointing to a possible defect in mitochondrial biogenesis. Furthermore, mitochondria isolated from cells treated with hsp90 inhibitors stimulate nuclear condensation in cell-free apoptosis assays.

Unlike most of the known cytoplasmic clients of hsp90, we demonstrate that mitochondrial proteins accumulate in cancer cells treated with hsp90 inhibitors. Mitochondrial protein upregulation occurs at a post-transcriptional level, and similar changes in mitochondrial ultrastructure and mitochondrial protein expression are exhibited in cancer cells treated with proteasome inhibitors. Furthermore, we demonstrate that the F1F0-ATPase OSCP subunit undergoes hsp90-dependent ubiquitination and has an increased protein half-life in hsp90-inhibited cells. This process of organellar protein turnover is reminiscent of endoplasmic reticulum associated degradation (ERAD), which functions both as a quality control and a regulatory pathway for ER proteins [13], [14].

Hsp90-regulated degradation of mitochondrial proteins represents a novel and unexpected pathway in overall cellular economy. Accumulation of mitochondrial proteins leading to intra-organellar protein folding stress may be an early and important event in cells treated with hsp90 or proteasome inhibitors for triggering mitochondrial apoptosis pathways.

## Results

### Hsp90 inhibition triggers mitochondrial protein accumulation

**Figure 1:**
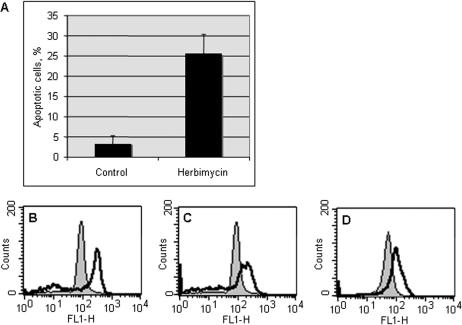
Colo-205 cells treated with Hsp90 inhibitors exhibit increased mitochondrial mass and apoptotic cell death. (A) Apoptosis measured by Annexin V/ PI staining at 72 h of herbimycin A treatment (0.5 µM). B) Mitochondrial mass assessed by NAO staining for (B) 17-AAG (0.4 µM, 24 h), (C) HA (0.5 µM, 48 h), (D) radicicol (1.5 µM, 72 h). Control (filled) vs. treated (empty) histograms.

**Figure 2:**
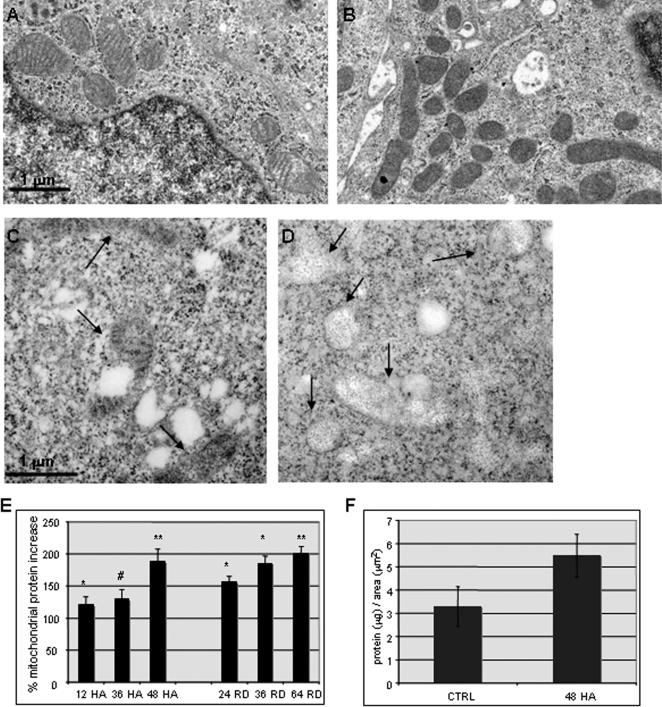
Mitochondrial protein accumulation with hsp90 inhibition. Transmission electron micrographs of control (A) and treated (B) COLO 205 cells (HA, 0.5 µM, 48 h) demonstrating increased number of mitochondrial profiles with dense matrix substance. Ultrathin sections from HA-treated COLO 205 cells (0.5 µM, 48 h) with no protease treatment (C) and after pronase digestion (D). Arrows indicate mitochondrial profiles. (E) Protein quantitation in mitochondrial fractions from 10^6^ COLO 205 cells treated with HA (0.5 µM) or radicicol (1.5 µM), normalized to untreated cells; average of three experiments, standard error, ^#^ *P* = 0.079, * *P*<0.05, ** *P*<0.005 for comparison to untreated cells. (F) Mitochondrial protein/10^6^ cells normalized to average total area of mitochondrial profiles/cell for control and HA-treated (0.5 µM, 48 h) COLO 205 cells. (*P*<0.05).

COLO 205 colon cancer cells treated with hsp90 inhibitors, including herbimycin A (HA), 17-allylamino-17-demethoxygeldanamycin (17-AAG) and radicicol, undergo terminal differentiation and apoptotic cell death within 72–96 h, preceded by dramatic changes to the mitochondrial compartment or chondriome [11] (Fig. 1 A and data not shown). Both the number of mitochondrial profiles and mitochondrial mass per cell, assessed by flow cytometry of cells stained with nonyl acridine orange, increased at 24–72 h of treatment with hsp90 inhibitors (Fig. 1 B–D and data not shown). Electron micrographs (EM) demonstrate that, in hsp90 inhibitor-treated cells, the mitochondrial matrix becomes progressively opacified with electron-dense material, and by 48 h the internal cristal membranes are completely obscured (Fig. 2A, B).

Increased mitochondrial matrix density can be due to a loss of water and ions (condensed conformation), but also may represent increased matrix protein content [15]. Mild pronase treatment of EM sections removed the electron-dense matrix material, consistent with its proteinaceous nature (Fig. 2C, D). Mitochondrial fractions were prepared for protein quantitation at various times after addition of hsp90 inhibitor. Total mitochondrial protein (normalized to cell number) increased two-fold after 48 h of treatment with HA or radicicol (Fig. 2E). Mitochondrial protein normalized to mitochondrial volume per cell (estimated by the summed area of EM mitochondrial profiles) also appeared to increase after HA treatment **(** Fig. 2F **)**.

**Figure 3:**
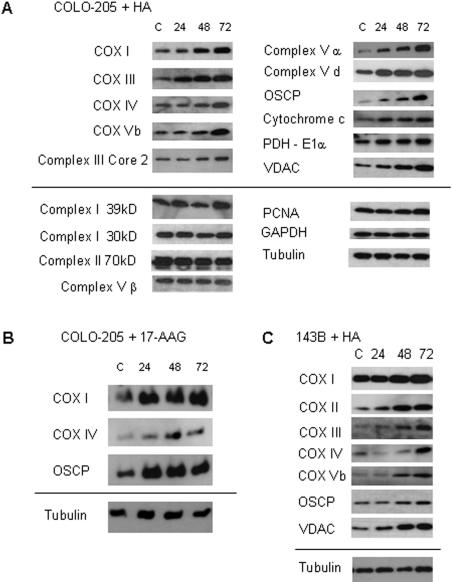
Changes in specific mitochondrial protein expression in response to hsp90 inhibition. Western blots from control and treated cells at 0, 24, 48 and 72 h. 4×10^4^ cell equivalents were loaded in each lane. COLO-205 cells treated with HA (0.5 µM) (A) or 17-AAG (0.4 µM ) (B). (C) 143B cells treated with HA (0.5 µM). PCNA, GAPDH and α-tubulin were used as loading controls. For each panel, proteins above the line are upregulated with hsp90 inhibitor treatment, and proteins below the line have stable expression.

We next determined whether expression of individual mitochondrial proteins changed in cells treated with hsp90 inhibitors (Fig. 3A). Immunoblots demonstrated that subunits of cytochrome c oxidase (COX I, III, IV, Vb), F1F0-ATPase (Complex V α, d, OSCP), and Complex III (Core 2), as well as cytochrome c, were upregulated with HA treatment. Mitochondrial proteins targeted to the outer mitochondrial membrane (VDAC) and matrix (pyruvate dehydrogenase E1α) also accumulated in HA-treated cells. Several electron transport chain subunits (Complex I 39 and 30 kD, Complex II 70 kD, and Complex V β) were not induced, indicating that hsp90 inhibition produced selective changes to the mitochondrial proteome (Fig. 3A). Protein expression levels of three non-mitochondrial proteins, proliferating cellular nuclear antigen (PCNA), glyceraldehyde-3-phosphate dehydrogenase (GAPDH) and tubulin, did not change in HA-treated cells. Similar increases in mitochondrial protein expression were observed with another hsp90 inhibitor, 17-AAG (Fig. 3B) and a second cell line, 143B osteosarcoma cells (Fig. 3C).

**Figure 4:**
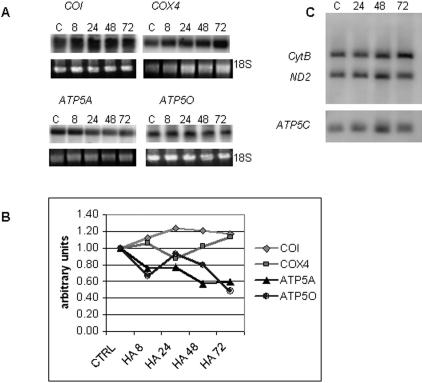
Hsp90 inhibitors affect mitochondrial protein expression at a post-transcriptional level. (A) Northern blots of mtDNA gene *COI* and nuclear genes *COX4*, *ATP5A*, and *ATP5O* in COLO 205 cells treated with HA (0.5 µM). 18S rRNA was used as loading control. (B) Quantitation of Northern blots by densitometry normalized to 18S rRNA levels. (C) Southern blot hybridized with mtDNA (*CytB* and *ND2*) and nuclear DNA gene (*ATP5C*) probes.

To investigate the level of regulation of mitochondrial protein expression in hsp90 inhibitor-treated cells, we performed Northern blots for mRNAs representing nuclear and mtDNA-encoded mitochondrial proteins (Fig. 4A). Neither mRNAs for chromosomal genes (COX IV (*COX4*), ATPase α (*ATP5A*), and OSCP (*ATP5O*)) nor the mtDNA gene COX I (*COI*) encoding proteins upregulated by hsp90 inhibitors were induced by HA treatment (Fig. 4A, B). In addition, mtDNA copy number did not increase with HA treatment (Fig. 4C). Thus, hsp90 inhibitors increase expression of several mitochondrial proteins via post-transcriptional regulation.

### Ubiquitination and proteasomal degradation of mitochondrial proteins

**Figure 5:**
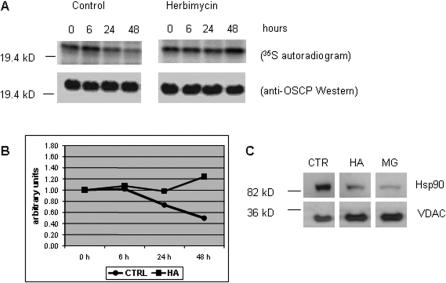
Hsp90 inhibition increases mitochondrial protein stability. (A) Immunoprecipitation of pulse-chase [^35^S]-labeled OSCP subunit from control and HA (0.5 µM) treated cells. HA was added to cells at the time of pulse labeling. Total OSCP protein is demonstrated by Western blotting in lower panel. (B) Densitometry of the OSCP autoradiography signal is graphed. (C) Mitochondrial localization of hsp90 in COLO 205 cells and response to HA (0.5 µM) and MG132 (0.5 µM) treatment for 48 h. Mitochondrial VDAC expression increases with both treatments.

We next examined mitochondrial protein synthesis and turnover in hsp90 inhibitor-treated cells. ^35^S-methionine/cysteine pulse-chase labeling followed by immunoprecipitation of the nuclear-encoded F1F0-ATPase OSCP subunit revealed an increase in protein half-life from 36–48 h in untreated cells to greater than 48 h in HA-treated cells (Fig. 5A, B). No differences were observed in initial ^35^S-labeling of OSCP between untreated and HA-treated cells. Therefore, the increase in mitochondrial OSCP protein expression following hsp90 inhibition is due to reduced protein turnover.

**Figure 6:**
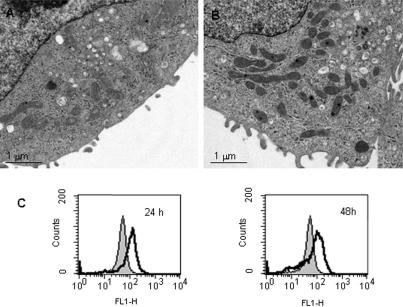
Proteasome inhibition results in increased mitochondrial mass and matrix condensation. Transmission electron micrograph of (A) control and (B) MG132-treated COLO 205 cells (0.5 µM MG132, 24 h). (C) Flow cytometric assay of mitochondrial mass by NAO staining. COLO 205 cells treated with MG132 (0.5 µM). Control (filled) vs treated (empty) histograms.

**Figure 7:**
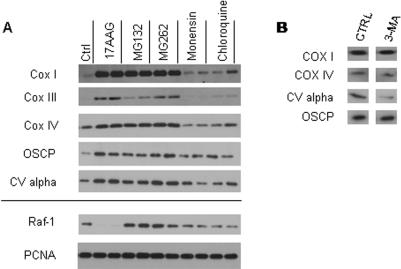
Mitochondrial protein expression in COLO 205 cells treated with Hsp90, proteasomal and lysosomal inhibitors. (A,B) Western blots of cell lysates after treatment with indicated inhibitors for 24 h. Inhibitor concentrations: 17-AAG (0.2, 0.4 µM), MG132 (0.25, 0.5 µM), MG262 (0.05, 0.1 µM), monensin (0.25, 0.5 µM), chloroquine (25, 50 µM), 3-MA (5 mM). Extracts from 2–4×10^4 ^cells were loaded per lane.

Hsp90 co-purifies with mitochondria in COLO 205 cells, but dissociates after treatment with hsp90 inhibitors (Fig. 5C). In addition to a direct role in stability of client proteins, hsp90 also functions in protein turnover mediated by the chaperone-dependent ubiquitin E3 ligase, CHIP [16]. To determine whether ubiquitin-dependent protein turnover affects the mitochondrial compartment, we treated COLO 205 cells with proteasome inhibitors. Condensed mitochondrial morphology and increased mitochondrial mass developed within 24 h of treatment with MG132, comparable to that observed with hsp90 inhibitors (Fig. 6). Immunoblot analysis showed increased expression of the same mitochondrial proteins (COX I, III, IV, OSCP, Complex V-alpha) with two different proteasomal inhibitors, MG132 and MG232, as seen with 17-AAG treatment (Fig. 7A). In contrast, Raf-1, a known hsp90 client protein, is degraded in cells treated with 17-AAG but accumulated in cells treated with proteasome inhibitors.

Hsp90 and proteasome inhibitors can cause ER protein folding stress and modulate the unfolded protein response (UPR) [17], [18]. To test whether mitochondrial protein accumulation is secondary to ER protein folding stress, we treated OSCP-GFP expressing 143B cells with tunicamycin, an inducer of the ER UPR. OSCP-GFP protein levels did not increase in cells treated wtih tunicamycin, while in combination with 17-AAG, tunicamycin reduced mitochondrial protein accumulation compared with 17-AAG alone ( Figure S1). We also tested inhibitors of autophagy for effects on mitochondrial protein expression in COLO 205 cells. Treatment with two different lysosomal inhibitors, monensin and chloroquine, or a macroautophagy inhibitor, 3-methyladenine (3-MA) did not increase mitochondrial protein expression at 24 h (Fig. 7 A, B).

**Figure 8:**
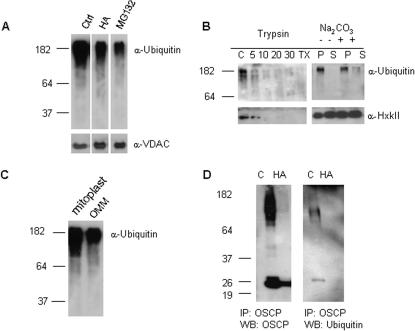
Mitochondria of COLO 205 cells contain ubiquitinated proteins. Anti-ubiquitin Western blots: (A) iodixanol gradient-purified mitochondria from control, HA- (0.5 µM, 48 h), and MG132-treated cells (0.5 µM, 24 h). VDAC signal is shown for loading control. (B) mitochondria after incubation with trypsin (5–30 min) or 0.1 M Na_2_CO_3_. TX-Triton X-100 (1%); P-pellet, S-supernatant, control blots of OMM-associated protein (hexokinase II); (C) OMM and mitoplast mitochondrial fractions after digitonin extraction. (D) Western blots of immunoprecipitated OSCP from COLO 205 cell extracts-control (C) and 48 h HA (0.5 µM) treatment.

We next investigated whether proteasomal functions were directly involved in turnover of mitochondrial proteins. Purified mitochondria were prepared from a 10,000×*g* pellet fraction of COLO 205 cell lysates by fractionation on a continuous iodixanol gradient (19–27%). Anti-ubiquitin immunoblotting recognized a high molecular weight smear in mitochondrial extracts from untreated cells, consistent with polyubiquitinated species (Fig. 8A). Mitochondria prepared from cells treated with HA or MG132 had reduced amounts of ubiquitinated proteins. Ubiquitin immunoreactive bands were sensitive to trypsin digestion of intact mitochondria, but mitochondrial association was retained in 0.1 M sodium carbonate (pH 11.5), suggesting that ubiquitinated proteins are either tightly bound to the mitochondrial surface or inserted in the outer mitochondrial membrane (OMM) (Fig. 8B). Digitonin extracts of OMM also contained high molecular mass ubiquitinated bands, although a greater proportion remained associated with the mitoplast fraction (Fig. 8C).

Immunoprecipitation of OSCP from cellular extracts recovered a broad smear at ∼80–180 kD size and a 30 kD band in addition to the expected 21 kD band representing mature OSCP. Subsequent immunoblotting with an anti-ubiquitin antibody recognized the 80–180 kD and 30 kD species, but not the 21 kD band, consistent with poly- and mono-ubiquitinated OSCP (Fig. 8D). OSCP immunoprecipitates from cells treated with HA exhibited only a 21 kD band, with no reactivity on anti-ubiquitin immunoblots. In view of the effect of proteasome inhibitors on OSCP protein expression, these results are consistent with ubiquitin-mediated OSCP turnover. Furthermore, hsp90 appears to be required for the attachment of ubiquitin to OSCP.

### Post-import proteasomal turnover of mitochondrial proteins

**Figure 9:**
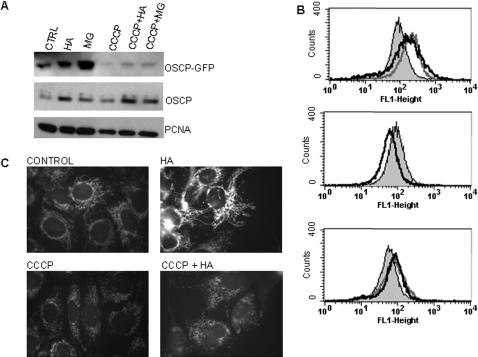
Hsp90 and proteasome inhibitors increase OSCP levels post-mitochondrial import. (A) Western blots of 143B cells transfected with OSCP-GFP and treated with HA (0.5 µM), MG132 (0.5 µM), and CCCP (10 µM) for 18 h. (B) Flow cytometry histograms of GFP fluorescence after 18 h of treatment. Top panel, filled histogram: control; open histograms: HA (light grey), MG-132 (dark grey). Middle panel, filled histogram: control; open histogram: CCCP; Bottom panel, filled histogram: CCCP; open histograms: CCCP+HA (light grey), CCCP+MG-132 (dark grey). (C) Fluorescence micrographs of GFP fluorescence in cells treated with HA, CCCP or CCCP plus HA for 24 h.

OSCP is a component of the F1F0-ATP synthase complex localized to the mitochondrial matrix, physically separated from cytoplasmic ubiquitin ligases. To address whether imported mitochondrial OSCP was susceptible to proteasomal degradation, we stably expressed an OSCP-GFP fusion protein in 143B cells. OSCP-GFP is efficiently imported to the mitochondrial matrix [19]. Proteasome and hsp90 inhibitors increased expression of OSCP-GFP, as well as the endogenous OSCP protein (Fig. 9A). Monitoring of GFP fluorescence intensity by flow cytometry provided results similar to immunoblot analysis (Fig. 9B-top panel). To isolate effects on imported mitochondrial OSCP-GFP, we treated cells with the uncoupling agent CCCP to prevent new ΔΨ_M_-dependent import of matrix-targeted proteins prior to adding proteasome or hsp90 inhibitors. CCCP treatment of 143B-OSCP-GFP cells decreased GFP fluorescence, as expected [20]. Even so, treatment with MG132 or HA increased OSCP-GFP expression in cells pre-treated with CCCP, as shown by immunoblot and flow cytometry, with a mitochondrial localization determined by fluorescence microscopy (Fig. 9A–C). These results are consistent with hsp90- and proteasome-dependent degradation of the OSCP subunit after mitochondrial import.

**Figure 10:**
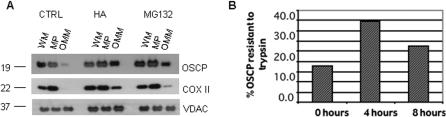
Recovery of inner mitochondrial membrane proteins OSCP and COX II in OMM fraction in cells treated with hsp90 and proteasome inhibitors. Western blots of whole mitochondria (WM), digitonin-extracted OMM and mitoplast (MP) fractions from COLO 205 cells treated with HA (0.5 µM, 48 h) and MG132 (0.5 µM, 24 h). (B) Immunoprecipitable [^35^S]-pulse-labeled mitochondrial OSCP after trypsin treatment of mitochondria, representative of two experiments.

In an established model of organellar protein turnover by ubiquitin-mediated proteasomal degradation, endoplasmic reticulum-associated degradation (ERAD), endoplasmic reticulum proteins are exported to the cytoplasm in a process termed retro-translocation. We questioned whether a similar process could operate for mitochondrial proteins. ERAD retro-translocation intermediates can be trapped by inhibition of downstream proteasomal processing [21]–[24]. We analyzed the localization of OSCP in mitochondrial membrane fractions before and after treatment with proteasome inhibitors. Sub-mitochondrial fractionation demonstrated the expected distribution for OSCP in mitoplasts (inner mitochondrial membrane and matrix) isolated from control cells (Fig. 10A). The abundant outer mitochondrial membrane protein VDAC is incompletely extracted by digitonin, presumably due to its association with contact sites between inner and outer mitochondrial membranes [25]. However, in cells treated with HA or MG132, a substantial proportion of the mature 21 kD OSCP is associated with the OMM. Notably, removal of the OSCP transit peptide occurs in the mitochondrial matrix, implying that export is required for the appearance of the 21 kD OSCP protein in the OMM fraction. A smaller amount of COX II appears in the OMM fraction from treated cells, suggesting that mtDNA-encoded proteins may also be exported.

In order to detect dynamic retro-translocation, we pulse-labeled cells with ^35^S-methionine/cysteine and examined mitochondrial import of OSCP by immunoprecipitation from mitochondrial fractions. In parallel, mitochondria were treated with trypsin to degrade OSCP associated with the OMM. The percentage of trypsin-resistant ^35^S-labeled mitochondrial OSCP increased during the first 4 h of chase, reflecting mitochondrial import of newly synthesized protein (Fig. 10B). However, after an additional 4 h chase period, the percentage of OSCP that was trypsin-sensitive increased in mitochondrial fractions, consistent with retro-translocation of previously imported protein.

### Regulation vs quality control

**Figure 11:**
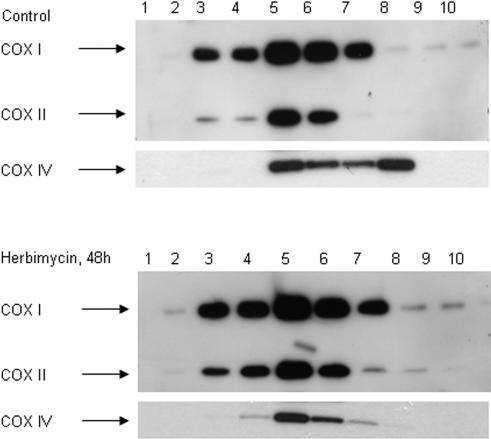
Improved cytochrome *c* oxidase assembly in HA-treated cells. Western blot of sucrose gradient density fractions of mitochondrial protein complexes; fraction 1 to 10 decreases in density.

Ubiquitin-mediated proteasomal degradation of ER proteins provides a mechanism for regulating protein expression as well as quality control of protein folding. To examine the effect of hsp90-regulated mitochondrial protein turnover on assembly of a multi-protein electron transport complex in COLO 205 cells, we solubilized mitochondrial membranes in dodecyl maltoside detergent and analyzed complex assembly by centrifugation on a sucrose density gradient [26]. Immunoblots were performed on collected fractions for individual cytochrome *c* oxidase subunits (Fig. 11). In untreated COLO 205 cells, COX I and COX II exhibit a broad peak centered at fraction 5, while COX IV has two peaks, one in fraction 5 coincident with COX I/II, and a second peak at lower density in fraction 8. In cells treated with hsp90 inhibitor, a unimodal distribution of COX IV is observed, with disappearance of the low density peak. These results suggest that inhibition of hsp90-regulated turnover of mitochondrial proteins can promote assembly of respiratory chain complexes, consistent with a regulatory function. Although oxygen consumption in COLO-205 cells treated with HA is only ∼35% of control cells, maximal respiration rates in the presence of the uncoupler CCCP, are identical for treated and untreated cells, suggesting that the total respiratory capacity is maintained for a significant period, in agreement with the complex assembly data (Figure S2).

**Figure 12:**
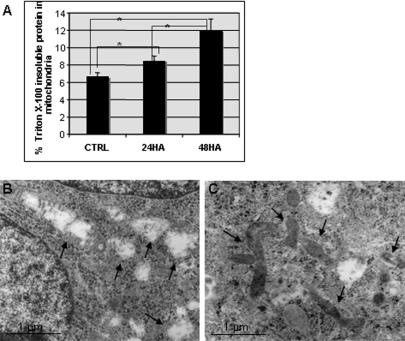
Mitochondrial proteins in HA-treated cells are resistant to detergent extraction. (A) Recovery of mitochondrial protein in lysis buffer with 0.5% Triton X-100 compared to CHAPS-Urea (HA 0.5 µM). * *P*<0.05. (B, C) Electron micrographs of mitochondria following glutaraldehyde pre-fixation and Triton X-100 extraction for control (B) and HA-treated COLO 205 cells (C). Mitochondria are indicated by arrows.

In its quality control function, ERAD extracts microsomal proteins as part of the UPR. We observed a consistent reduction in the amount of mitochondrial protein extractable with nonionic detergents in cells treated with hsp90 inhibitors (Fig. 12A). Taking into account the increase in total (SDS-extractable) mitochondrial protein in HA-treated cells, the amount of Triton X-100 insoluble mitochondrial protein increased more than three-fold after 48 h of HA treatment.

We surmised that the dense mitochondrial matrix observed in electron micrographs of cells treated with proteasome or hsp90 inhibitors could represent aggregated or unfolded protein. In order to examine mitochondrial protein solubility in situ, we lightly fixed cells prior to extraction in low concentrations of non-ionic detergent, followed by processing for electron microscopy. Control COLO 205 cells exhibited empty mitochondrial profiles consistent with complete extraction of mitochondrial matrix proteins with up to 1 h of pre-fixation (Fig. 12B). In contrast, mitochondrial matrix proteins in HA-treated cells were completely resistant to Triton X-100 extraction with as little as 15 min of pre-fixation (Fig 12C). Thus, one consequence of mitochondrial protein accumulation in cells treated with hsp90 inhibitors is a change in protein solubility, with potentially negative consequences for mitochondrial function.

## Discussion

Hsp90 inhibitors induce apoptosis, with selectivity for cancer cells, via mitochondrial pathways. Analysis of the mitochondrial compartment, using mitochondrially targeted fluorescent probes and electron microscopy, revealed early increases in mitochondrial mass, associated with distinctive ultrastructural changes, in cells treated with hsp90 inhibitors. To investigate the basis of these changes, we analyzed mitochondrial protein expression. We identified an increase in total mitochondrial protein, normalized to cell number, as well as multiple individual mitochondrial proteins in COLO 205 and 143B cells treated with hsp90 inhibitors, including nuclear-encoded (COX IV, Vb, F1F0-ATPase α, d, OSCP, cytochrome c, VDAC1, PDH E1α) and mtDNA-encoded respiratory complex subunits (COX I, III). Notably, several other respiratory complex subunits examined were unaffected. We excluded transcriptional regulation for several of these proteins by the stable steady state levels of cognate cytosolic and mitochondrial mRNAs and mtDNA copy number. Unexpectedly, *ATP5A* and *ATP5O* mRNAs declined after 48 and 72 h of HA treatment (Fig. 4). This may represent a retrograde response (mitochondria-to-nuclear signaling) to the increased mitochondrial mass.

Pulse-chase studies demonstrated that the mitochondrial F1F0-ATPase OSCP subunit is stabilized in hsp90-inhibited cells. Treatment with proteasome inhibitors MG132 and MG262, but not lysosomal (monensin, chloroquine) or autophagy (3-methyladenine) inhibitors, produced a similar pattern of mitochondrial protein upregulation in COLO 205 cells, suggesting that proteasomal protein degradation pathways are involved in mitochondrial protein turnover. We next demonstrated that immunoprecipitation of OSCP from purified mitochondrial lysates isolated several slower migrating bands recognized by anti-ubiquitin immunoblots and consistent with mono- and poly-ubiquitinated OSCP, in addition to the mature 21 kD OSCP. This result is consistent with direct proteasomal degradation of a mitochondrial protein, mediated via poly-ubiquitin conjugation. Furthermore, hsp90 inhibition suppressed the recovery of ubiquitin-modified OSCP in mitochondrial lysates, suggesting hsp90 is required for effective interaction of mitochondrial OSCP with one or more ubiquitin ligases.

The ubiquitin-proteasome system is not previously known to participate in the turnover of mitochondrial proteins. However, several proteomic efforts to catalogue ubiquitin-modified proteins have identified mitochondrial proteins including respiratory chain subunits residing in the inner mitochondrial membrane and matrix (see Table 1). A recent analysis of the mouse heart ubiquitinated proteome shows that proteins localized in mitochondria represent a major fraction (38 %) and expands the list of the ubiquitin conjugated mitochondrial proteins [33]. Proteasomal turnover of the mitochondrial heme synthetic enzyme 5-aminolevulinic acid synthase 2 was demonstrated [34]. The prominent 30 kD band consistent with monoubiquitinated OSCP, could represent an intermediate product in polyubiquitination, or carry out a separate role in protein trafficking, lysosomal degradation, or protein interactions [35].

**Table 1:** Mitochondrial proteins identified as ubiquitin conjugates in previous studies.

	Protein	Protein	Hu	Sc	Ar	Biological Process	Reference
1	NADH dehydrogenase (ubiquinone)	NDI1		X		oxidative phosphorylation	[27]
2	Succinate dehydrogenase cytochrome B560 subunit	C560_HUMAN	X			oxidative phosphorylation	[28]
3	Succinate dehydrogenase cytochrome b	SDH3		X		oxidative phosphorylation	[27]
4	Succinate dehydrogenase (ubiquinone) activity (Sdh1p homolog)	YJL045W		X		oxidative phosphorylation	[27]
5	Ubiquinol cytochrome c reductase complex Core 2 Protein	UCR2_HUMAN	X			oxidative phosphorylation	[28]
6	8.5 kDa subunit of the ubiqunol-cytochrome c oxidoreductase complex	QCR10		X		oxidative phosphorylation	[27]
7	Ubiquinol cytochrome-c reductase subunit 8	QCR8		X		oxidative phosphorylation	[27]
8	Ubiquinol-cytochrome c reductase	AT4G32470.1			X	oxidative phosphorylation	[29]
9	Cytochrome c1	CYT1		X	X	oxidative phosphorylation	[27], [29]
10	Subunit VII of cytochrome c oxidase	COX7		X		oxidative phosphorylation	[27]
11	Cytochrome oxidase assembly factor	COX15		X		oxidative phosphorylation	[27]
12	Modulates cytochrome c oxidase activity	COX13		X		oxidative phosphorylation	[27]
13	F1F0-ATP synthase alpha subunit	ATP1	X	X		oxidative phosphorylation	[28], [30], [31]
14	F1F0-ATP syntahse beta subunit	ATPB_HUMAN	X			oxidative phosphorylation	[28], [30]
15	F1F0-ATP synthase B chain, mitoch. precursor	ATPF_HUMAN	X			oxidative phosphorylation	[28]
16	F1F0-ATP synthase gamma chain, mitochondrial	ATPC			X	oxidative phosphorylation	[29]
17	Protein associated with mitochondrial ATP synthase	TIM11		X		oxidative phosphorylation	[27]
18	Cytochrome c	CYC_HUMAN	X			oxidative phosphorylation	[28]
19	Iso-2-cytochrome c	CYC7		X		oxidative phosphorylation	[27]
20	Plant uncoupling mitochondrial protein	PUMP			X	oxidative phosphorylation	[29]
21	Malate dehydrogenase precursor	MDHM_HUMAN	X		X	tricarboxylic acid cycle	[28], [29], [30]
22	Isocitrate dehydrogenase precursor	IDHA_HUMAN	X			tricarboxylic acid cycle	[30]
23	Dihydrolipoamide dehydrogenase (E3) precursor	DLDH_ HUMA	X			tricarboxylic acid cycle	[28], [30]
24	Aconitase hydratase	ACON_HUMAN	X			tricarboxylic acid cycle	[30]
25	Citrate synthase	CIT1		X		tricarboxylic acid cycle	[27]
26	Alpha-ketoglutarate dehydrogenase	KGD1		X		tricarboxylic acid cycle	[27]
27	Alpha subunit of succinyl-CoA ligase	LSC1	X			tricarboxylic acid cycle	[27]
28	Beta subunit of succinyl-CoA ligase	LSC2	X			tricarboxylic acid cycle	[27]
29	Pyruvate dehydrogenase E1 component alpha subunit	AT1G59900.1			X	tricarboxylic acid cycle	[29]
30	Pyruvate dehydrogenase E1 component beta subunit	AT5G50850.1			X	tricarboxylic acid cycle	[29]
31	2-oxoglutarate dehydrogenase E1	AT3G55410.1			X	tricarboxylic acid cycle	[29]
32	Acetyl-CoA carboxylase	ACC1		X		fatty acid biosynthesis	[27], [31]
33	Trifunctional enzyme	FAS2		X		fatty acid biosynthesis	[27], [31]
34	Aspartate aminotransferase precursor	AATM_HUMAN	X			aminoacid metabolism	[30]
35	Required for the translation of OLI1 mRNA.	AEP2		X		protein biosynthesis	[27]
36	Mitochondrial and cytoplasmic valyl-tRNA synthetase	VAS1		X		valine-tRNA ligase	[27]
37	5-aminolevulinate synthase	HEM1		X		hem metabolism	[27]
38	Protein involved in mitochondrial iron accumulation	MMT2		X		iron metabolism	[27]
39	Oxodicarboxylate carrier	ODC1		X		mitochondrial transport	[27]
40	The major mitochondrial ADP/ATP translocator	PET9		X		mitochondrial transport	[27], [31]
41	Mitochondrial substrate carrier family protein	AT4G01100.1			X	mitochondrial transport	[29]
42	Phosphate carrier protein, mitochondrial precursor	MPCP_HUMAN	X			mitochondrial transport	[28]
43	Voltage-dependent anion selective channel protein 2	POR2_HUMAN	X		X	mitochondrial transport	[28], [29]
44	Translocase of the outer mitochondrial membrane	TOM5		X		mitochondrial translocation	[27]
45	Translocase of the outer mitochondrial membrane	TOM7		X		mitochondrial translocation	[27]
46	Translocase of outer mitochondrial membrane, 70 kDa	OM70_HUMAN	X	X		mitochondrial translocation	[27]
47	Mitochondrial import receptor subunit Tom22 homolog	OM22_HUMAN	X			mitochondrial translocation	[28]
47	Translocase of the inner membrane	TIM50			X	mitochondrial translocation	[27]
49	Mitochondrial 28S ribosomal protein S33	RT33_HUMAN	X			mitochondrial nucleic acid metabolism	[28]
50	Mitochondrial elongation factor	EFGM_ARATH			X	mitochondrial nucleic acid metabolism	[29]
51	Mitochondrial transcription termination factor	AT5G23930.1			X	mitochondrial nucleic acid metabolism	[29]
52	Cytochrome b reductase	CBR1		X		electron transport	[27]
53	Hypothetical ORF	NDE1		X		ethanol fermentation	[27]
54	Hypothetical ORF	NDE2		X		ethanol fermentation	[27]
55	Involved in receptor-mediated endocytosis and mitochondrial organization	DNM1		X		mitochondrion organization and biogenesis	[27]
56	Protein involved in mitochondrial fusion	FZO1		X		mitochondrion organization and biogenesis	[27], [31]
57	Putative hemolysin-like protein with three transmembrane domains	MAM3		X		mitochondrion organization and biogenesis	[27]
58	Mitochondrion organization and biogenesis	MDM38		X		mitochondrion organization and biogenesis	[27]
59	Mitochondrion inheritance	MDM10		X		mitochondrion inheritance	[27]
60	Cytoskeleton organization and biogenesis	MDM20		X		cytoskeleton organization and biogenesis	[27]
61	Stress-70 (mitochondrial)	GR75_HUMAN	X			chaperone	[28]
62	Prohibitin	PHB_HUMAN	X		X	chaperone	[29], [32]
63	Smac protein, mitochondrial precursor	SMAC_HUMAN	X			apoptosis	[28]

Hu–*Homo Sapiens*; Sc.–*Saccharomyces cerevisiae*; Ar–*Arabidopsis Thaliana*.

Proteasome and hsp90 inhibitors increased the expression and mitochondrial fluorescence of an OSCP-GFP fusion protein, even in the presence of a protonophore causing collapse of mitochondrial membrane potential, ΔΨ_M_. Mitochondrial import of matrix-targeted proteins, including OSCP, is ΔΨ_M_-dependent, implying that these inhibitors affect the turnover of previously imported mitochondrial OSCP. Ubiquitin-conjugated proteins in mitochondrial fractions are tightly associated with the outer mitochondrial membrane as shown by digitonin extraction, protease susceptibility, and resistance to alkali extraction. These results are analogous to protein ubiquitination in endoplasmic reticulum associated degradation (ERAD), where both soluble and membrane inserted ER proteins undergo retro-translocation before ubiquitin conjugation at the cytosolic face of the ER [13], [14].

Mitochondrial subfractionation demonstrated a shift in localization of mature OSCP to the OMM in cells treated with hsp90 or proteasome inhibitors. This likely represents a retro-translocation intermediate, as observed for several ERAD substrates [21]–[24], and suggests that hsp90 function and proteasome activity are required, at least in part, for complete retro-translocation. Hsp90 co-purified with COLO 205 mitochondria, although the association was diminished in HA- and MG132-treated cells. TRAP1, a mitochondrial paralog of hsp90, is also inhibited by the tested hsp90 inhibitors [36], [37]. Knockdown of TRAP1 expresssion using TRAP1 short-hairpin RNAs did not affect responses to 17-AAG, although basal expression levels for several mitochondrial proteins were diminished consistent with the postulated role of TRAP1 as an intramitochondrial chaperone (Figure S3).

Hsp90 is reported to function in protein degradation, in addition to its role in protein folding, via its association with CHIP, an E3 ubiquitin ligase [16]. Reduction of CHIP protein expression by 60–90% by shRNA-mediated RNA interference did not result in increased mitochondrial protein expression or suppress responses to 17-AAG (Figure S3), raising the possibility of alternative E3-ligases, perhaps recognizing processed mitochondrial proteins as N-end rule substrates, as hsp90 co-factors. Hsp90 may also facilitate transport of ubiquitin-conjugated OSCP to the proteasome. Hsp90 inhibition leads to an increase in insoluble ubiquitinated cytosolic proteins [38].

Mitochondrial proteins can also be degraded in a nonselective manner via autophagy, or more specifically, mitophagy. Autophagic degradation of organelles and bulk cytosolic components is physiologically activated during nutrient starvation to maintain an adequate supply of nutrients for cell survival. Inhibition of autophagy with 3-MA or inhibition of lysosomal activity with monensin or chloroquine did not lead to increased mitochondrial protein expression in COLO 205 cells grown in standard media. In contrast, 3-MA increased OSCP-GFP expression in cells grown under nutrient starvation conditions [39] (Figure S4). Our data suggests that hsp90 and the 26S proteasome are components of an alternative pathway for selective degradation of mitochondrial proteins.

Increased mitochondrial protein expression in cells treated with hsp90 inhibitors was associated with improved cytochrome oxidase assembly, but ultimately led to an increase in detergent-insoluble mitochondrial proteins, and mitochondrial dysfunction [11]. Analogous to defective protein folding in the endoplasmic reticulum, a mitochondrial unfolded protein response may play a role in the apoptotic effects of hsp90 and proteasome inhibitors [40].

## Materials and Methods

### Cell lines

COLO 205 cells were grown as described [11]. 143B cells were grown in High Glucose-Dulbecco's Minimal Essential Medium with 10% fetal bovine serum in 10% CO_2_. Amino-acid deprived medium was cysteine/methionine-free HG- DMEM. 143B cells were transfected with pEGFP-N1 vector (BD Biosciences) bearing a full length human OSCP cDNA in frame with a COOH-terminal GFP sequence, using Fugene6 Transfection Reagent (Roche). Individual clones were obtained after selection in 100 µg/ml G418.

### Reagents and Antibodies

All chemicals were purchased from Sigma-Aldrich with the exception of MG262 and Tunicamycin (Calbiochem). Herbimycin A, 17-AAG and radicicol (Sigma) were dissolved in DMSO at 1 mg/ml and stored in aliquots at −70°C. MG132 and MG262 were prepared as 1 mM stocks in DMSO and stored at −70°C. Monensin was prepared as a 1 mM stock in ethanol, and chloroquine (50 mM) and 3-MA (250 mM) were dissolved in H_2_0 and stored at 4°C. Tunicamycin was dissolved in DMSO (20 mM) and stored at −20°C. Monoclonal antibodies against mitochondrial respiratory chain proteins were obtained from MitoSciences Inc. Other antibodies used: VDAC (PC548, Oncogene), pyruvate dehydrogenase E1alpha subunit (A-21323, Invitrogen), cytochrome c (556433, BD Biosciences), c-Raf (610151, BD Biosciences), CHIP/STUB1 (IMG-3137, Imgenex), PCNA (NA03, Oncogene), GAPDH (ab8245, Abcam), tubulin (CP06-100UG, Oncogene), hexokinase (sc-6521, Santa Cruz Biotechnology), ubiquitin (sc-8017, Santa Cruz; 550944, BD Biosciences), Hsp90 (sc-7947, Santa Cruz).

### Flow cytometry

For nonyl acridine orange (NAO) staining, cells were resuspended in phosphate-buffered saline (PBS) at 1×10^6^ cells/ml and incubated with 1 nM NAO for 10 min at room temperature in the dark. Cells were analyzed on a FACScan cytometer (Becton Dickinson). Annexin V binding and propidium iodide (PI) staining was performed according to manufacturer's protocol (Annexin V:FITC Apoptosis Detection Kit II, BD Biosciences).

### Electron microscopy

Transmission electron microscopy was performed as described [41]. To address the nature of the dense mitochondrial matrix material, ultrathin sections of cell pellets embedded in water-soluble media were incubated with 1 mg/ml pronase at 37°C for 1 h. Cells were pre-fixed for various times (15 min, 30 min or 1 h) in 0.25% glutaraldehyde, exposed to 0.25% Triton X-100 in PBS for 15 min and post-fixed in half strength Karnovsky's fixative. Transmission electron micrographs were taken using a Jeol 1010 transmission electron microscope operating at 80 kV. The 2D area of mitochondrial profiles was calculated from transmission electron micrographs using Image J (NIH). Profiles were traced in sets of 10 cells (6,000x magnification) for control and HA-treatment. The mitochondrial area was summed for each cell to derive an average total mitochondrial area per cell, calculated in µm^2^ after correction by the magnification factor.

### Western blotting

Whole cell lysates were prepared from 5×10^6^ cells/ml resuspended in lysis buffer (1% n-dodecyl β-D maltoside, 25 mM Hepes pH 7.2, 200 mM NaCl, 5 mM EDTA plus protease inhibitors) on ice for 30 min. Soluble proteins were separated from insoluble material by centrifugation at 18,000x *g* for 20 min at 4°C. Protein extracts were denatured in 1X Laemmli buffer supplemented with 10 mM dithiothreitol (DTT) at 37°C for 30 min, loaded on SDS-polyacrylamide gels and separated by electrophoresis in Tris-Glycine buffer at 100 V. Proteins were transferred to 0.2 µΜ PVDF membranes (Millipore) in CAPS buffer for 2 h at 200 mA or overnight at 40 V. Membranes were blocked in 5% fat free milk in phosphate-buffered saline with 0.05% Tween-20 (PBST) for 1 h at room temperature, incubated with primary antibody overnight at 4°C and with secondary antibodies (goat anti-mouse or goat anti-rabbit conjugated to horseradish peroxidase) for 2 h at room temperature. Signal was developed by chemiluminescence (ECL Plus, GE Healthcare).

### Southern and Northern blot analysis

Total genomic DNA extracted from cells was digested with NcoI (Promega), separated with 1X TAE (40 mM Tris, 20 mM acetic acid, 1 mM EDTA) running buffer on a 0.7% agarose gel, and transferred to nitrocellulose (Zeta-Probe, Bio-Rad). Southern blots were hybridized to [α-^32^P]-labeled probes generated by random priming. Final wash steps were performed with 0.1xSSC and 0.1% SDS at 65°C. RNA isolation and Northern analysis was performed as described [41]. I.M.A.G.E. clones used to obtain probes for Southern and Northern blotting: *ND2*: 5581341, *CytB*: 4257731, *COI*: 5139968, *COX4*: 68531, *ATP5A*: 3355758, *ATP5C*: 647125, *ATP5O*: 5551187. Southern blots were performed in duplicate and Northern blots in triplicate. Hybridization signals from a representative Northern blot were quantitated using ImageJ (NIH) and normalized to 18S rRNA levels.

### Mitochondrial preparations

Cells were disrupted either by nitrogen cavitation (500 psi for 10 min) or 10–15 passages through a 25G needle in homogenization buffer (0.25 M sucrose, 1 mM EGTA, 10 mM HEPES, 0.5 % bovine serum albumin, pH 7.4, with protease inhibitors). Mitochondrial pellets were obtained from post-nuclear supernatant by centrifugation at 10,000×*g*. For some studies, mitochondria were purified by centrifugation on a continuous 19–27% iodixanol gradient (Optiprep) for 2 h at 70,000×*g*. Alkali extraction was performed with 0.1 M Na_2_CO_3_ for 30 min on ice; trypsin digestion (50 µg/ml) was performed at 37°C. Outer mitochondrial membrane (OMM) preparations were obtained by digitonin (1 mg/mg protein) extraction for 20 min. Sucrose gradient fractionation of respiratory chain complexes was performed as described [26].

### Immunoprecipitation

The F1F0-ATPase OSCP subunit was immunoprecipitated from cells lysed in TENT buffer (0.5% Triton X-100, 50 mM Tris-HCL, 150 mM NaCL, 5 mM EDTA, pH 7.4 with freshly added protease inhibitors) at 4°C. After centrifugation at 12,000×*g* for 15 min to remove insoluble material, the extracts were incubated overnight at 4°C with anti-OSCP antibody cross-linked to Protein G Sepharose beads (Amersham Biosciences), washed three times with TENT buffer, and eluted with 100 mM glycine, pH 2.8. Eluted proteins were neutralized with 1 M Tris pH 8.0, denatured at 65°C for 10 min in 1X Laemmli buffer with 10 mM fresh DTT, and separated by 4–15% gradient SDS-PAGE for Western blotting.

For pulse-chase studies, cells were plated 24 h before labeling with 50 µCi/ml [^35^S]-methionine/cysteine (Trans label, ICN) in met/cys-free medium. For trypsin sensitivity of mitochondrial OSCP, cells were pulse-labeled for 2 h and then chased in the presence of cycloheximide 100 µg/ml. Mitochondrial suspensions were prepared at each time point (from 8×10^6^ cells). One-half of each sample was incubated with trypsin 50 µg/ml for 20 min at 37°C, followed by immunoprecipitation of OSCP from TENT extracts of trypsin-treated and untreated mitochondria. Gels were fixed for 30 min at room temperature in 40% methanol, 10% acetic acid, and 5% glycerol, followed by incubation in Amplify Fluorographic Reagent (GE Healthcare), gel drying and exposure to phosphor storage screens (Molecular Dynamics). Autoradiograph images (Typhoon 8600, GE Healthcare) were analyzed using ImageQuant software.

### Differential solubility of mitochondrial proteins

Mitochondrial fractions (50 µg protein) were extracted in TENT buffer for 30 min at 4°C. Soluble proteins were recovered by centrifugation for 30 min at 20,000×*g*. The insoluble pellet was dissolved in CHAPS-Urea (8M urea, 2% CHAPS, 150 mM NaCl, 5 mM EDTA, 10 mM Tris pH 7.4) at room temperature. Protein concentration of TENT-soluble and insoluble fractions was measured by Bradford assay (Bio-Rad).

### Fluorescence microscopy

143B cells expressing OSCP-GFP fusion protein were grown on glass coverslips and treated with 0.5 µM HA, 20 µM CCCP, or both for 24 h. Cells were fixed in 4% paraformaldehyde and mounted on slides for fluorescence imaging (Nikon Eclipse E800, 100X Plan Fluor objective). Photomicrographs of cells subjected to different treatments were acquired during the same experimental session with identical exposure times.

### RNA interference

Short-hairpin sequences against CHIP (STUB1) and TRAP1 genes were cloned into the lentiviral vector pLKO.1-TRC (Addgene). The target sequences selected are: CHIP sh1 5′- GCACGACAAGTACATGGCGGA -3′[42]; CHIP sh3: 5′- GAAGAGGAAGAAGCGAGACAT-3′ (The RNAi Consortium #TRCT0000001576); TRAP1 sh1: 5′- ACCGTCCATGTTTGATGTGAG-3′; TRAP1sh2: 5′- GTACAGCAACTTCGTCAGC-3′ [30]; TRAP1sh3: 5′-GCGCTCATCAAGAAGCTGAAT-3′ (The RNAi Consortium #TRCN0000060676).

Viral particles were obtained by co-transfection of 293T cells with the pLKO.1-shRNA, envelope (pMD2G) and packaging (pCMVdR8.74) vectors. 143B and OSCP-GFP 143B cells were transduced with the shRNA lentivirus and selected in puromycin at 1 µg/ml. Control cells were transduced with virus carrying a scrambled shRNA sequence (pLKO.1-scramble shRNA; Addgene). CHIP and TRAP1 mRNA expression levels were determined by quantitative real-time PCR using the Brilliant SYBR Green Master Mix (Stratagene). Expression was normalized to actin. CHIP protein expression was confirmed by Western blotting.

### Oxymetry

Colo 205 cells were harvested with trypsin, resuspended in respiration buffer (125 mM KCl, 2 mM K_2_HPO_4_, 1 mM MgCl_2_, 5 mM K-Hepes, pH 7, 1 mM EGTA, 5 mM glutamate, 5 mM malate) at 2×10^7^ cells/ml and allowed to equilibrate for 5 min before loading in the electrode chamber (Oxytherm, Hansatech Instruments). Cells were stirred at 37°C during O_2_ consumption measurements. After obtaining a stable respiration rate, CCCP (10 µM) was added to uncouple respiration and determine maximal O_2_ consumption rates.

### Statistics

Statistical analysis was done using Student's paired *t*-test. The data are expressed as means±standard error and statistical significance was indicated by *P*<0.05.

## Supporting Information

Figure S1 Endoplasmic reticulum stress induced by tunicamycin does not cause OSCP-GFP accumulation. Flow cytometry histograms of OSCP-GFP fluorescence after 24 h of treating 143-B cells with tunicamycin (A) 20 µM; (B) 100 µM; (C) 17-AAG (0.4 µM); (D) tunicamycin (100 µM) and 17-AAG (0.4 µM).
(0.04 MB TIF)

Figure S2 Oxygen consumption rates of control COLO 205 and herbimycin A (0.5 µM) treated-COLO 205 cells at 48 h. Coupled versus uncoupled (10 µM) respiration. *P*<0.005 for coupled respiration.
(0.04 MB TIF)

Figure S3 Downregulation of TRAP1 or CHIP expression does not affect the response to 17-AAG. Quantitative real-time PCR data for CHIP and TRAP1 mRNA expression in control 143B cells, cells transduced with scrambled, CHIP (A) or TRAP1 (B) shRNA. Expression is normalized to β-actin levels. (C) Western blot of CHIP protein expression in control cells and cells transduced with CHIP shRNA1, CHIP shRNA3 or scrambled shRNA. (D) Western blots showing accumulation of OSCP and COXIII protein with 17-AAG treatment in control and shRNA expressing cells. Tubulin was used as loading control. Flow cytometry histograms of GFP expression with cells expressing scrambled shRNA (E), TRAP1 shRNA3 (F), CHIP shRNA1 (G), CHIP shRNA3 (H). Control-gray profile; 17-AAG treatment (0.4 µM, 24 hours)-black profile.
(0.08 MB TIF)

Figure S4 Regulation of OSCP-GFP protein levels by hsp90 and proteasome inhibitors in complete and amino acid-deficient media. Flow cytometry histograms of GFP fluorescence in 143B cells transfected with OSCP-GFP. Cells were treated with 17-AAG (0.4 µM), MG-132 (1 µM), or 3-MA (5 mM) for 24 h. The autophagy inhibitor 3-MA has an effect on mitochondrial protein expression only in amino acid-deficient media (methionine/cysteine-free medium).
(0.06 MB TIF)
