# Supplementary figures and images for: Hsp90 Inhibition Decreases Mitochondrial Protein Turnover
Source: PLoS One. 2007 Oct 24;2(10):e1066. doi: 10.1371/journal.pone.0001066 (PMC2031825; doi:10.1371/journal.pone.0001066)

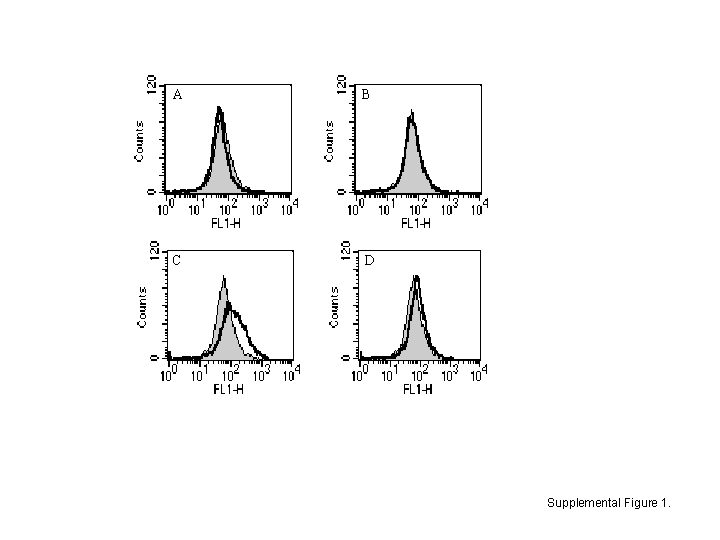

Supplement: Figure S1 — Endoplasmic reticulum stress induced by tunicamycin does not cause OSCP-GFP accumulation. Flow cytometry histograms of OSCP-GFP fluorescence after 24 h of treating 143-B cells with tunicamycin (A) 20 µM; (B) 100 µM; (C) 17-AAG (0.4 µM); (D) tunicamycin (100 µM) and 17-AAG (0.4 µM). (0.04 MB TIF) [file pone.0001066.s001.tif]

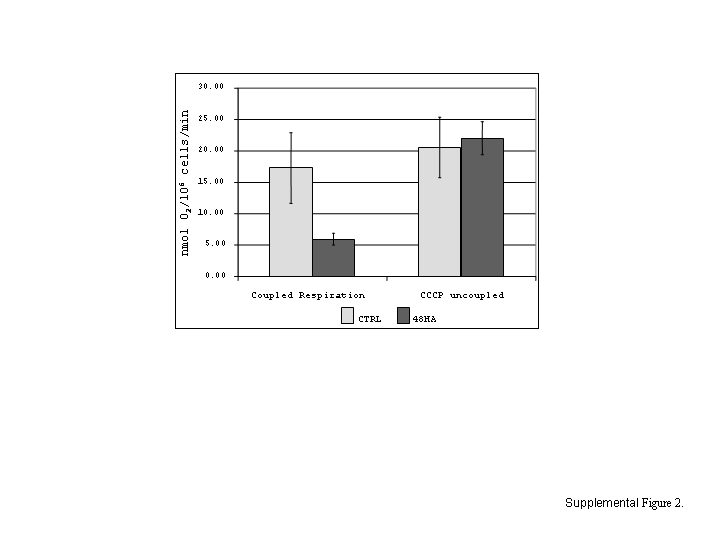

Supplement: Figure S2 — Oxygen consumption rates of control COLO 205 and herbimycin A (0.5 µM) treated-COLO 205 cells at 48 h. Coupled versus uncoupled (10 µM) respiration. P<0.005 for coupled respiration. (0.04 MB TIF) [file pone.0001066.s002.tif]

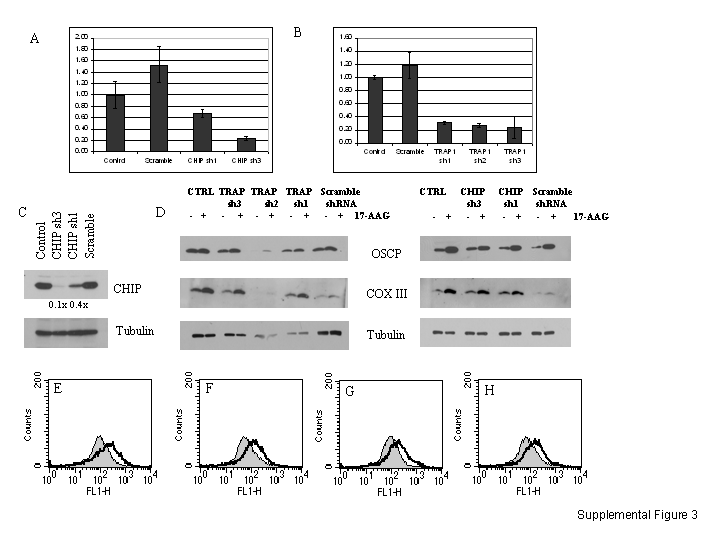

Supplement: Figure S3 — Downregulation of TRAP1 or CHIP expression does not affect the response to 17-AAG. Quantitative real-time PCR data for CHIP and TRAP1 mRNA expression in control 143B cells, cells transduced with scrambled, CHIP (A) or TRAP1 (B) shRNA. Expression is normalized to β-actin levels. (C) Western blot of CHIP protein expression in control cells and cells transduced with CHIP shRNA1, CHIP shRNA3 or scrambled shRNA. (D) Western blots showing accumulation of OSCP and COXIII protein with 17-AAG treatment in control and shRNA expressing cells. Tubulin was used as loading control. Flow cytometry histograms of GFP expression with cells expressing scrambled shRNA (E), TRAP1 shRNA3 (F), CHIP shRNA1 (G), CHIP shRNA3 (H). Control-gray profile; 17-AAG treatment (0.4 µM, 24 hours)-black profile. (0.08 MB TIF) [file pone.0001066.s003.tif]

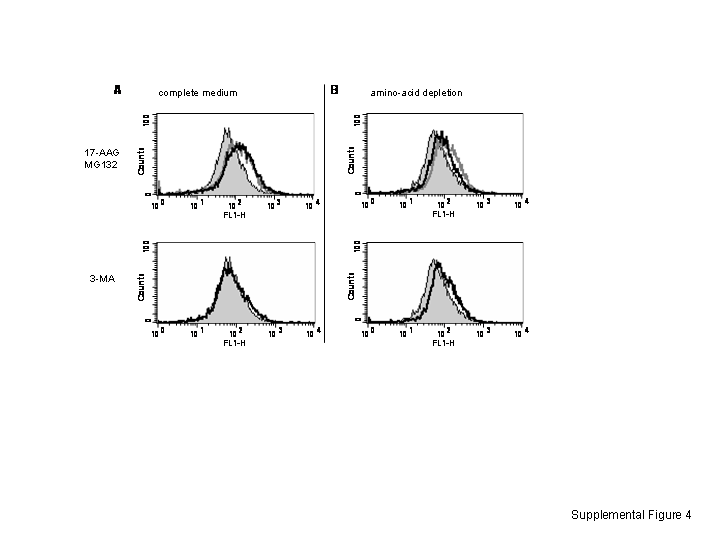

Supplement: Figure S4 — Regulation of OSCP-GFP protein levels by hsp90 and proteasome inhibitors in complete and amino acid-deficient media. Flow cytometry histograms of GFP fluorescence in 143B cells transfected with OSCP-GFP. Cells were treated with 17-AAG (0.4 µM), MG-132 (1 µM), or 3-MA (5 mM) for 24 h. The autophagy inhibitor 3-MA has an effect on mitochondrial protein expression only in amino acid-deficient media (methionine/cysteine-free medium). (0.06 MB TIF) [file pone.0001066.s004.tif]
